# The Williams-Beuren Syndrome—A Window into Genetic Variants Leading to the Development of Cardiovascular Disease

**DOI:** 10.1371/journal.pgen.1002479

**Published:** 2012-02-02

**Authors:** Gregory N. Adams, Alvin H. Schmaier

**Affiliations:** Departments of Medicine and Pathology, Case Western Reserve University, Cleveland, Ohio, United States of America; University of Oxford, United Kingdom

## Background

**Figure 1:**
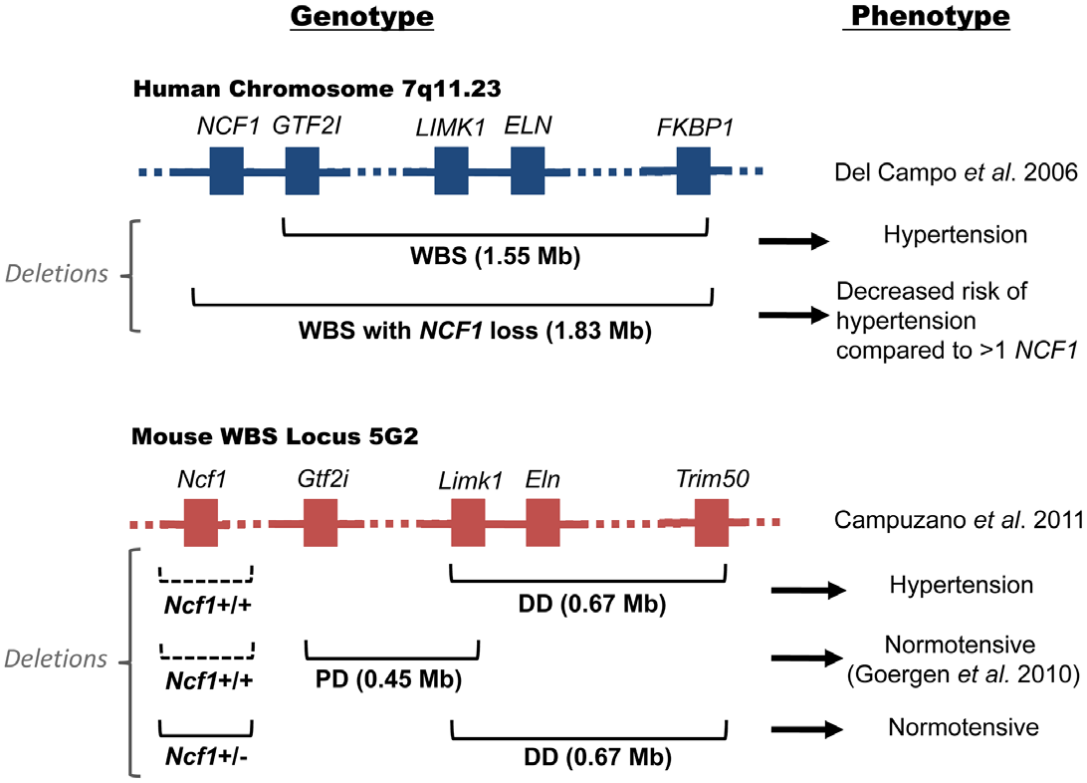
Genotype and phenotype of human and murine Williams-Beuren Syndrome. Top: human WBS locus; Bottom: murine WBS locus; Left: genotypes; Right: phenotypes. In the human WBS locus, deletion of *ELN* is associated with hypertension. WBS patients with deletions incorporating loss of *NCF1* (1.83 Mb) are at lower risk for hypertension compared to patients with the more common WBS deletion (1.55 Mb). In the murine WBS locus, the DD mouse with a 0.67 Mb deletion containing *Eln* is hypertensive. It contains both *Ncf1* alleles (dotted lines indicate that the gene is present). Mice with an adjacent PD deletion are normotensive [4]. Hypertension in the DD mouse is corrected when mated with *Ncf1^+/−^* mice.

Williams-Beuren Syndrome (WBS) arises when there is a genomic microdeletion at human chromosome 7q11.23 (Mouse 5G2), resulting in various cardiovascular, developmental, metabolic, and mental disorders [1]. Cardiovascular complications from WBS are a frequent cause of death. The deleted region is predisposed to non-allelic homologous recombination (NAHR) due to the presence of repetitive DNA regions called segmental duplications. WBS can result in deletions of up to 1.83 Mb in a region containing roughly 28 genes that includes the gene *ELN* encoding the tissue structural protein elastin [2]. Many of the cardiovascular features of WBS can be partially explained by elastin defects. WBS individuals have a combined prevalence of cardiovascular disease of 84% that includes supra- and sub-aortic stenosis (SVAS); aortic, pulmonary, and mitral valvular disease; aortic coarctation; hypertension; and, less commonly, myocardial infarction [2], [3]. These defects have also been found in mouse models [4]. Elastin not only provides “elastic” support for vessels, it also serves as a negative regulator for smooth muscle cell proliferation. WBS patients are also at high risk for hypertension and *Eln^+/−^* mice are hypertensive [1], [4]. In 2006, Del Campo et al. recognized that hemizygosity of the *NCF1* gene as a result of the largest recognized WBS microdeletion—about 1.83 Mb—decreases the risk for hypertension in WBS patients compared to those possessing the more common smaller deletion (1.55 Mb) not incorporating *NCF1* (Figure 1, top) [5]. *NCF1* codes for p47*^phox^*, a critical subunit in the assembly of NADPH oxidase (NOX); homozygous deficiency accounts for 20% of patients with chronic granulomatous disease, a disorder associated with repeated infections due to an inability to kill bacteria. p47*^phox^* is a major effector of angiotensin II (AngII), as demonstrated by a lack of elevation in blood pressure of *Ncf1* ^−/−^ mice [6]. In this issue of *PLoS Genetics*, Campuzano and colleagues replicate the WBS cardiovascular phenotype in a WBS mouse model with and without a deletion of the *Ncf1* gene [7]. Their study has broad implications for our understanding of hypertension- and reactive oxygen species (ROS)-related cardiovascular disease.

## A Genetic Basis for Hypertension in WBS

The investigation of Campuzano et al. [7] determined whether oxidative stress is a feature of WBS mice and whether genetic or pharmacologic reduction of NOX activity reduces oxidative stress and ameliorates hypertension associated with these mice. Vascular NOX production of superoxide has an important role in various cardiovascular pathologies, including hypertension. Superoxide antagonizes the vasculo-protective molecule nitric oxide (NO) either through direct interaction with NO or through oxidation of the endothelial NO-synthase (eNOS) enzyme co-factor tetrahydrobiopterin. Campuzano et al. observed that WBS mice called “DD”, with a 0.67 Mb deletion from *Limk1* to *Trim50* (that contains *Eln*), manifest a cardiovascular phenotype including hypertension with elevated angiotensinogen (*Agt*), renin (*Ren*), and angiotensin converting enzyme (*Ace*) mRNA throughout life (Figure 1, bottom) [7]. Another WBS syndrome mouse called “PD” with a 0.45 Mb deletion from *Gtf2i* to *Limk1* (not containing *Eln*) is normotensive (Figure 1, bottom) [4]. These data suggest that in WBS the absence of the elastin gene is necessary for hypertension. However, the DD condition was reversed when the mice were mated with *Ncf1* ^+/−^ mice, indicating that the presence of the *Ncf1* gene also contributes to the observed hypertension. As predicted, AngII levels in the DD/*Ncf1* ^+/−^ mice were reduced with respect to the DD mice alone, but elevated with respect to wild type mice. These combined studies indicate that both the *Eln* gene deletion and the *Ncf1* gene presence contribute to observed murine hypertension. It is of note that vascular pathologies such as supravalvular aortic stenosis were also corrected in DD mice partially depleted of the *Ncf1* gene.

## Role of ROS in Hypertension

In addition to elevated AngII levels, DD mice have elevated protein nitrosylation and superoxide anions as determined by dihydroethidium fluorescence in the ascending aorta. In contrast, DD/*Ncf1* ^+/−^ mice have reduced ROS in their aorta. These observations are consistent with other rodent hypertensive phenotypes [8], [9]. The fact that genetic reduction of ROS-producing mechanisms occurred in the DD/*Ncf1* ^+/−^ mice suggested that pharmacologic treatment to reduce ROS may also be helpful in treating DD mouse hypertension. Treatment of DD mice with the broad antioxidant apocynin and losartan (an angiotensin receptor type 1 antagonist) independently lowered blood pressure in DD mice and, together, their effect was additive. Blood pressure control was associated with reduced vessel ROS and lowered plasma AngII levels. Further, the anti-ROS therapies reduced the degree of anatomical changes in cardiac hypertrophy and vascular elastic fiber fragmentation. These pharmacologic studies suggest an alternative approach to treating the hypertensive phenotype in WBS patients. Currently, the most common treatments for WBS-induced hypertension are beta-blockers and calcium channel blockers. Using the present WBS mouse studies as an insight, the combined use of a specific antioxidant with losartan appears mechanistically more rational.

## Use of Antioxidants in Hypertension

The use of antioxidants for the treatment of vascular oxidant stress associated with cardiovascular disease has been questioned after failed clinical trials using non-specific antioxidants such as vitamins C and E [10]. Recognizing specific etiologies of superoxide in hypertension suggests that past antioxidant clinical trials were not well targeted to superoxide or hydrogen peroxide. To date, ROS-specific treatments such as the superoxide dismutase mimetic tempol have not been used in clinical trials. Tempol has been partially successful in animal models to treat hypertension [8], [11], [12]. When tempol is tagged with a mitochondrial-specific tag, the drug mitoTEMPO has been used to successfully treat hypertension in two rodent models [8], [9]. Campuzano et al. [7] also used the antioxidant apocynin to treat hypertension and both pre- and post-natal SVAS in the WBS mouse model. Although the specificity of apocynin for NOX is controversial, when it is delivered clinically by inhalation, it lowers an exhaled breath marker for ROS and increases a nitric oxide marker [13], [14].

## ROS in Thrombosis

In addition to hypertension and developmental structural defects in the cardiovascular system, increased vascular ROS may increase arterial thrombosis risk. Several animal models have been associated with increased vascular ROS and higher arterial thrombosis risk. Both heme oxygenase I–deleted mice and prolylcarboxypeptidase-deficient mice have increased vascular ROS and reduced arterial thrombosis occlusion times [9], . Vascular ROS is associated with uncoupled eNOS, inactivated thrombomodulin, and increased endothelial cell tissue factor and plasminogen activator inhibitor 1 [9], [16]. In the case of prolylcarboxypeptidase deficiency, in vivo treatment with apocynin or tempol resulted in correction of thrombosis risk and reduction in tissue ROS. Myocardial infarction and stroke are uncommon in WBS patients, probably due to the overriding risk posed by their severe cardiovascular developmental abnormalities. However, we would predict that the DD mice would be prothrombotic on ROS-generating thrombosis models, and specific antioxidants to superoxide or hydrogen peroxide would reduce any thrombosis risk, in addition to ameliorating hypertension.

## Future Directions

The studies by Campuzano et al. [7] on WBS show a double-hit genetic basis for ROS- and hypertension-related cardiovascular disease. They indicate that both pharmacologic and genetic targets could be used to manage the specific manifestations of WBS, and hypertension in general. Further, we believe that these approaches may also be useful in reducing arterial thrombosis risk in susceptible populations.
